# NAC and MYB Families and Lignin Biosynthesis-Related Members Identification and Expression Analysis in *Melilotus albus*

**DOI:** 10.3390/plants10020303

**Published:** 2021-02-05

**Authors:** Lijun Chen, Fan Wu, Jiyu Zhang

**Affiliations:** State Key Laboratory of Grassland Agro-Ecosystems, Key Laboratory of Grassland Livestock Industry Innovation, Ministry of Agriculture and Rural Affairs, College of Pastoral Agriculture Science and Technology, Lanzhou University, Lanzhou 730020, China; chenlj16@lzu.edu.cn (L.C.); wuf15@lzu.edu.cn (F.W.)

**Keywords:** *Melilotus albus*, NAC and MYB genes, expression analysis, promoter region, lignin biosynthesis

## Abstract

*Melilotus albus* is an annual or biennial legume species that adapts to extreme environments via its high stress tolerance. NAC and MYB transcription factors (TFs) are involved in the regulation of lignin biosynthesis, which has not been studied in *M. albus*. A total of 101 *MaNAC* and 299 *MaMYB* members were identified based on *M. albus* genome. Chromosome distribution and synteny analysis indicated that some genes underwent tandem duplication. Ka/Ks analysis suggested that *MaNAC*s and *MaMYB*s underwent strong purifying selection. Stress-, hormone- and development-related cis-elements and MYB-binding sites were identified in the promoter regions of *MaNACs* and *MaMYBs*. Five *MaNAC*s, two *MaMYB*s and ten lignin biosynthesis genes were identified as presenting coexpression relationships according to weighted gene coexpression network analysis (WGCNA). Eleven and thirteen candidate *MaNAC* and *MaMYB* genes related to lignin biosynthesis were identified, respectively, and a network comprising these genes was constructed which further confirmed the *MaNAC* and *MaMYB* relationship. These candidate genes had conserved gene structures and motifs and were highly expressed in the stems and roots, and qRT-PCR further verified the expression patterns. Overall, our results provide a reference for determining the precise role of *NAC* and *MYB* genes in *M. albus* and may facilitate efforts to breed low-lignin-content forage cultivars in the future.

## 1. Introduction

Transcription factors (TFs) play an important role in plant growth and development. NAC (no apical meristem (NAM), *Arabidopsis* transcription activation factor (ATAF1/2), and cup-shaped cotyledon (CUC2)) family members are plant-specific TFs. NAC TFs have a highly conserved N-terminal region and a variable C-terminal region; the N-terminal regions share a conserved DNA-binding region, whereas the C-terminal regions have various transcriptional regulatory domains [1,2,3]. In addition, the C-terminal region of some NAC proteins has α-helical transmembrane motifs that are related to stress responses [4,5]. These features help these proteins repress or activate various downstream genes to regulate multiple molecular or cellular processes. NAC proteins are involved in the regulation of various biological functions. Overexpression of STRESS-RESPONSIVE NAC 1 significantly enhanced the drought resistance of transgenic rice in the field, with no phenotypic changes or yield penalty [6]. ATAF1, which encodes an NAC TF, is a stimulus-dependent attenuator of abscisic acid signaling involved in mediating penetration resistance [7].

The MYB gene family is one of the largest TF families. MYB proteins are characterized by a highly conserved DNA-binding domain that generally consists of up to four imperfect amino acid sequence repeats (R) of 50–53 amino acids, each encoding three α-helices [8]. The second and third helices of each repeat form a helix–turn–helix structure with regularly interspersed triplet tryptophan residues forming a hydrophobic core [9]. The functions of many AtMYBs have been studied in *Arabidopsis*. AtMYB5 and AtMYB23 control trichome extension and branching [10], and AtMYB5 also regulates seed coat development [11]. AtMYB33 and AtMYB65 redundantly facilitate anther development [12]. MYB96 mediates both ABA-auxin crosstalk in the drought stress response and lateral root growth, and overexpressing MYB96 in *Arabidopsis* enhanced drought resistance, with reduced lateral roots [13].

Lignin is composed of phenylpropanoid-derived polymers and exists in the secondary cell walls of all vascular plants [14]. Lignin provides structural rigidity for plants to stand upright and strengthens the cell wall to withstand the negative pressure generated during transpiration [15]. It is widely accepted that lignin biosynthesis is controlled by the *NAC* and *MYB* gene regulatory network [16]. In the network, VASCULAR-RELATED NAC-DOMAIN 1–7 (VND1–7), NAC SECONDARY WALL THICKENING PROMOTING FACTOR 1–3 (NST1–3), and SECONDARY WALL-ASSOCIATED NAC DOMAIN PROTEIN 1 (SND1) act as the first layer of master switches, and MYB46 and MYB83, the downstream targets of the NAC proteins, function as the second layer of master switches to regulate downstream genes involved in secondary wall formation [17,18,19,20]. MYB58 and MYB63, which are regulated by SND1, are specific transcriptional activators of lignin biosynthesis to regulate secondary wall formation, and overexpression of MYB58 and MYB63 results in the activation of specific lignin biosynthesis genes [21]. Furthermore, the expression of MYB58 and MYB63 is regulated by MYB46 and homologs of SND1 (NST1, NST2, VND6, and VND7) [21]. These proteins can directly activate downstream lignin biosynthesis genes (PAL, C4H, and 4CL) [22].

*Melilotus* spp., which comprises 19 species, are important annual or biennial legume crop species [23]. The members of the *Melilotus* genus are highly stress tolerant and are adapted to extreme environments. Compared with traditional legume crop species, *Melilotus* species can grow in moderately saline soils while maintaining normal seed yields. *Melilotus* species are usually used as green manure crops, as a crop fertilizer (with a relatively high nitrogen fixation rate) and as a nectar source [24]. In addition, the nitrogen fixation ability and dry matter of *Melilotus* are significantly larger than those of *Medicago sativa*, and *Melilotus* produces yields of up to 640 kg of dry matter per mu, which is much greater than that produced by two-year-old *M. sativa* [25]. However, the lignin content of the stems of *Melilotus* is greater than that of *M. sativa*. Lignin is the key element that limits cell wall digestibility and limits the digestible energy available to ruminants [26]. *Melilotus* is widely distributed in tropical regions throughout both Asia and Europe. At present, the genetic diversity and population structure of *Melilotus* have been studied [27], interspecific phylogenetic relationships of the *Melilotus* genus have been identified [28,29], the standard barcode sequences of 18 *Melilotus* species have been constructed [30], the genetic improvement of *Melilotus albus* has been evaluated [31], and the transcriptome and small RNAs of *M. albus* have been analyzed [32,33]. However, the TF families in this species have not been thoroughly analyzed.

In this study, we conducted a genome-wide analysis of the NAC and MYB TF families in *M. albus*. A total of 101 MaNAC TFs and 299 MaMYB TFs were identified, and the gene structure, phylogenetics, promoter region, evolution and coexpression relationships were analyzed. Eleven and thirteen candidate MaNAC and MaMYB genes, respectively, that may regulate lignin biosynthesis were identified, and their networks were constructed. This genome-wide analysis of the MaNAC and MaMYB TF families might contribute to the future functional characterization of *M. albus* and lay a foundation for future studies on lignin biosynthesis. This research may provide a reference for molecular breeding to develop low-lignin-content forage cultivars of *M. albus*.

## 2. Results

### 2.1. Identification of NAC and MYB Genes

BLAST analysis of 155 OsMYB and 197 AtMYB coding sequences (CDSs) and protein sequences against the *M. albus* CDSs and protein sequences predicted approximately 400 sequences that contained Myb or Myb-like repeats. The domains of candidate MaMYB proteins were further examined via Pfam analysis. A total of 299 nonredundant *MaMYB* genes were identified and used for further analysis. The *MaMYB* genes were classified into three distinct subfamilies: R1-MYB genes (175), R2R3-MYB genes (123), and R1R2R3-MYB genes based on the number of Myb repeats (Table S1). Using similar methods, we identified 101 *MaNAC* genes that have NAM domains (Table S2).

### 2.2. Genome Distribution and Gene Synteny Analysis

**Figure 1:**
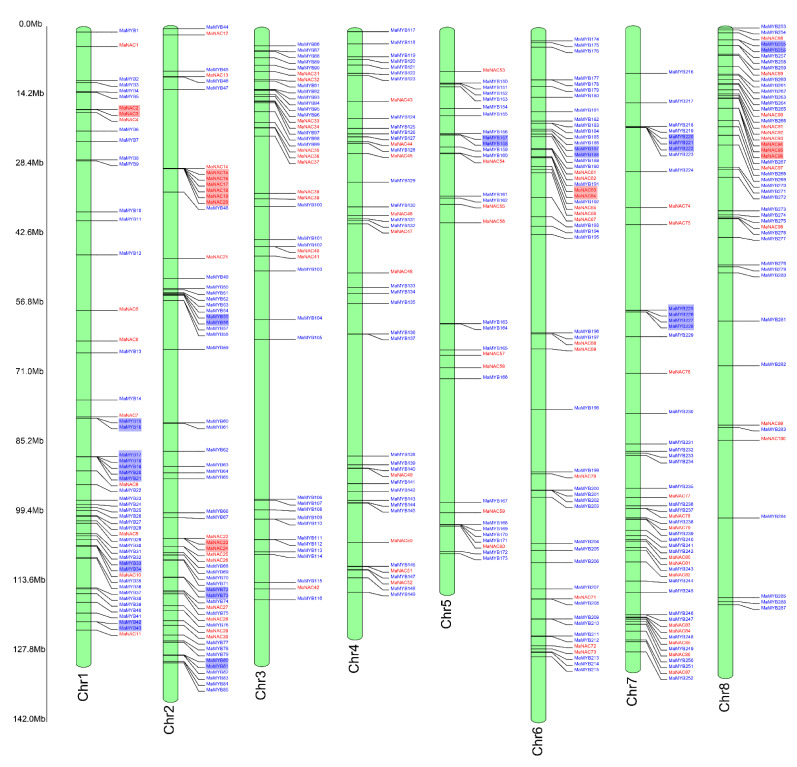
Chromosomal distribution of *MaNAC*s and *MaMYB*s. The red letters represent NAC transcription factors (TFs), and the blue letters represent MYB TFs. The red background represents tandem duplication genes of the NAC family. The blue background represents tandem duplication genes of the MYB family.

**Figure 2:**
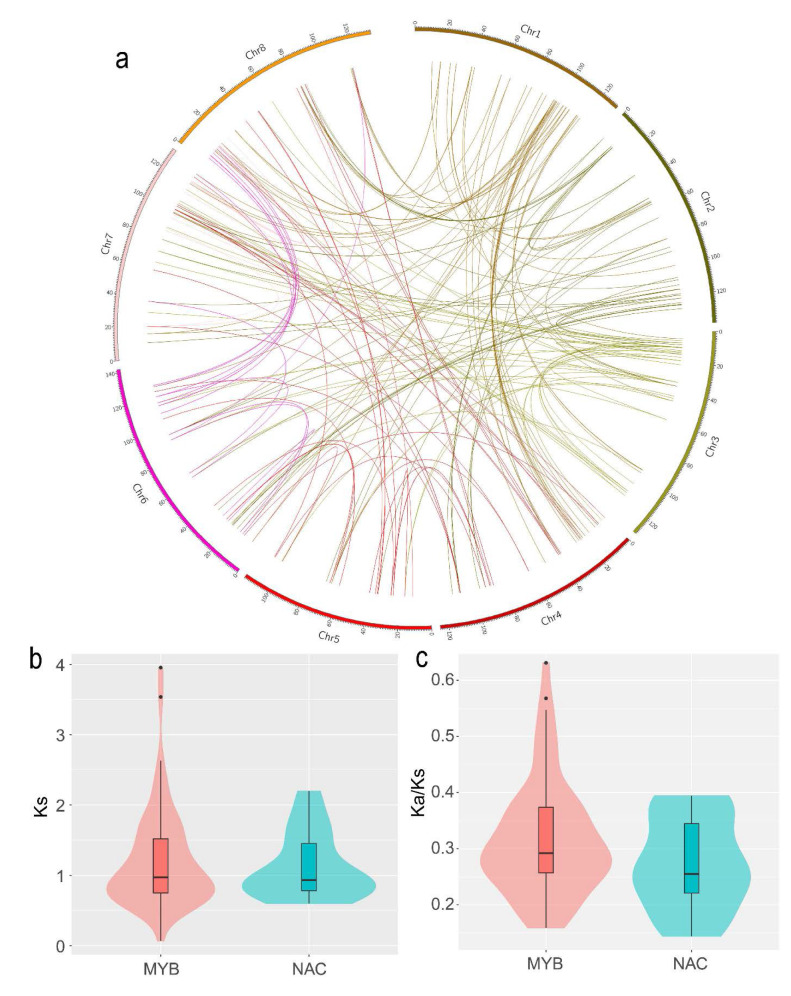
Synteny analysis of MaNAC and MaMYB gene pairs. (**a**) Synteny of MaNAC and MaMYB paralogs in M. albus. (**b**) Ks value of MaNAC and MaMYB paralogs. (**c**) Ka/Ks value of MaNAC and MaMYB paralogs.

Among all the NAC and MYB family members, 100 *MaNAC*s and 287 *MaMYB*s were mapped onto eight chromosomes (Figure 1). The distribution of *MaNAC*s and *MaMYB*s on the chromosomes was uneven. Chromosome 5 contained the lowest density, with eight *MaNAC*s and 24 *MaMYB*s (Figure 1). Chromosome 1 contained the largest number of genes, with 19 *MaNAC*s and 42 *MaMYB*s. In addition, the distribution of *NAC* and *MYB* genes at chromosomal loci revealed that 30 (10.03%) and 15 (14.85%), respectively, were the result of tandem duplications (Figure 1). Eight tandem repeats of *MaNAC* genes were present on chromosome 2, and 11 tandem repeats of *MaMYB* genes were present on chromosome 1. Five tandem duplication genes of *MaNAC* were found on chromosome 2. Three tandem duplication genes of *MaNAC* were found on chromosome 8. Three and three *MaMYB* tandem duplication genes were found on chromosomes 1 and 7, respectively (Figure 1). The synteny analysis showed a total of 48 NAC duplicated gene pairs and 136 MYB duplicated gene pairs in *M. albus* (Figure 2a, Table S3). Furthermore, some of the synteny gene pairs distributed in the same chromosome (Figure 2a). The tandem duplication genes and synteny gene pairs distributed in the same chromosome may be caused by segmental duplication [34]. The Ks could be used to evaluate the gene duplication evolution that had occurred after divergence from their ancestors. To estimate the selection pressure on the MaNACs and MaMYBs during the *M. albus* evolutionary, we calculated the Ka and Ks values and Ka/Ks ratio. The median Ks and Ka/Ks values of MYB was slightly larger than that of NAC (Figure 2b,c, Table S4). The results indicate that the gene duplication events of MYB occurred earlier than that of NAC. In addition, the Ka/Ks values of all gene pairs were less than 1, which indicated that the NAC and MYB genes of *M. albus* experienced strong purifying selection (Figure 2c).

### 2.3. Phylogenetic Analysis of NAC and MYB TF Genes

**Figure 3:**
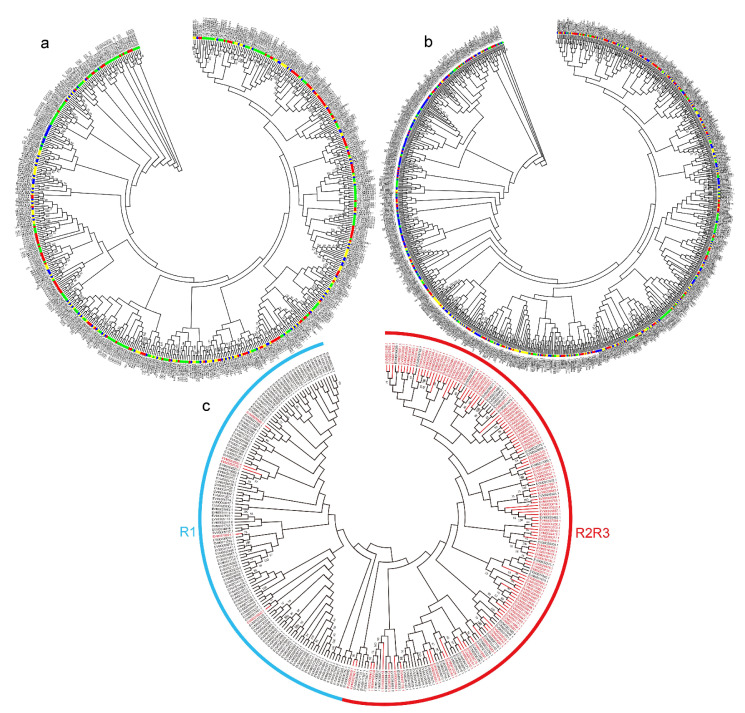
Phylogenetic analysis of NAC and MYB members. (**a**) Phylogenetic relationships of NAC proteins from *M. albus*, *M. truncatula*, Arabidopsis and rice. (**b**) Phylogenetic relationships of MYB proteins from *M. albus*, *M. truncatula*, Arabidopsis and rice. (**c**) Phylogenetic relationships of MaMYB proteins. The genes with the R2R3 domain are marked in red. All trees were constructed via MEGA 7.0 with the neighbor-joining (NJ) method (bootstrap, 1000 replicates).

**Figure 4:**
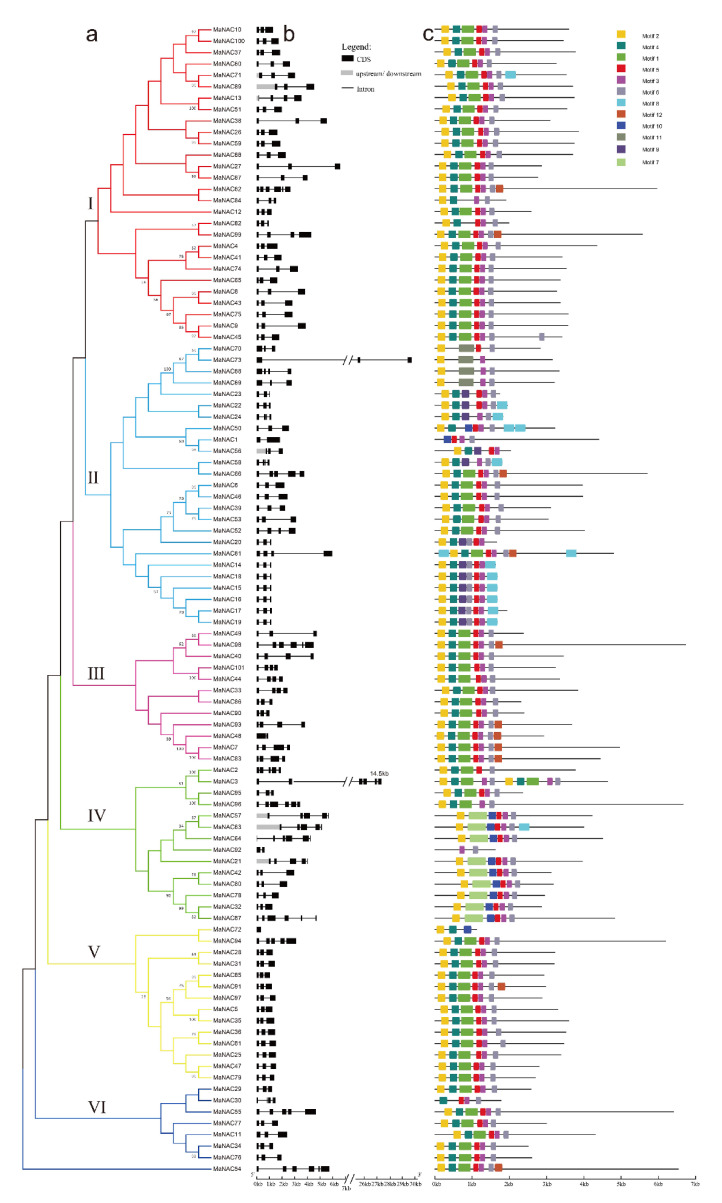
Phylogenetic relationships, gene structure and motif compositions of *M. albus* NAC members. (**a**) Phylogenetic tree of MaNAC proteins. The tree was constructed via MEGA 7.0 with the NJ method (bootstrap, 1000 replicates). Bootstrap values greater than 50% are shown on the nodes. (**b**) Intron/exon structures of *MaNAC* genes. (**c**) MaNAC protein motifs.

Phylogenetic comparative analysis of NACs and MYBs from different plant species revealed the conservation and diversification of the NAC and MYB TF family members. To examine the evolutionary relationships among the NAC or MYB gene family members from *M. albus*, *M. truncatula*, *Arabidopsis* and rice, a neighbor-joining (NJ) phylogenetic tree was constructed (Figure 3a,b). Most of the subgroups contained the NACs from *M. albus*, *M. truncatula*, *Arabidopsis* and rice, which suggested a common ancestor before the divergence of these species (Figure 3a). Some clades included only *M. albus* and *M. truncatula* members. One clade included only *M. albus* NACs, indicating that these *NAC* genes may have been acquired in *M. albus* after its divergence from the last common ancestor or that those genes had been lost either in rice, *M. truncatula* or *Arabidopsis*. The gain or loss of species-specific NACs led to functional divergence. Compared with the rice (155), *M. truncatula* (185) and *Arabidopsis* (197) MYB family, the *M. albus* (299) MYB family is substantially larger. *M. albus*, rice, *M. truncatula* and *Arabidopsis* had independent clades, and clades that included only *M. albus* MYB members were more abundant than those that included the other three species (Figure 3b). These results indicate that the NAC family was more conserved than the MYB family and that the MYB family members evolved faster. In the MaNAC protein phylogenetic tree, all proteins were divided into six clades—group I to group VI (Figure 4a). The phylogenetic tree of the MaMYB proteins showed that these proteins were clustered into two clades: one clade included mainly R2R3-MYB proteins, and the other clade included mainly R1-MYB proteins (Figure 3c). R2R3-MYB proteins with the conserved R2R3 sequences clustered into the same subgroup and may have a common evolutionary origin.

### 2.4. Gene Structure and Protein Motif Analysis

To better understand the similarity and diversity of gene structure among *MaNAC*s or *MaMYB*s, their intron/exon structure pattern was investigated by aligning their gene sequences and CDSs. With respect to the MaNAC TF family, 71 *MaNAC* genes had three exons, and only two *MaNAC* genes had one exon (Figure 4b). In addition, genes in the same subgroups generally contained similar intron and motif patterns (Figure 4b). For example, most of the genes in subgroups I, II and V had three exons. Thirteen motifs were identified in the *MaNAC*s. With the exceptions of *MaNAC1*, *MaNAC92*, and *MaNAC30*, nearly all the *MaNAC*s contained motif 2, and most *MaNAC*s contained motifs 1, 2, 3, 4 and 5. For the MaMYB TF family, 11 genes had one exon, but the number of exons varied from 1 to 12 (Figure S1). The motif structures were similar in one clade (Figure S2). The length of MYB protein motifs varied from 11 to 51 amino acids, and the number of motifs in each MYB varied from 2 to 10 (Figure S2).

### 2.5. Gene Promoter Region and Functional Analysis

**Figure 5:**
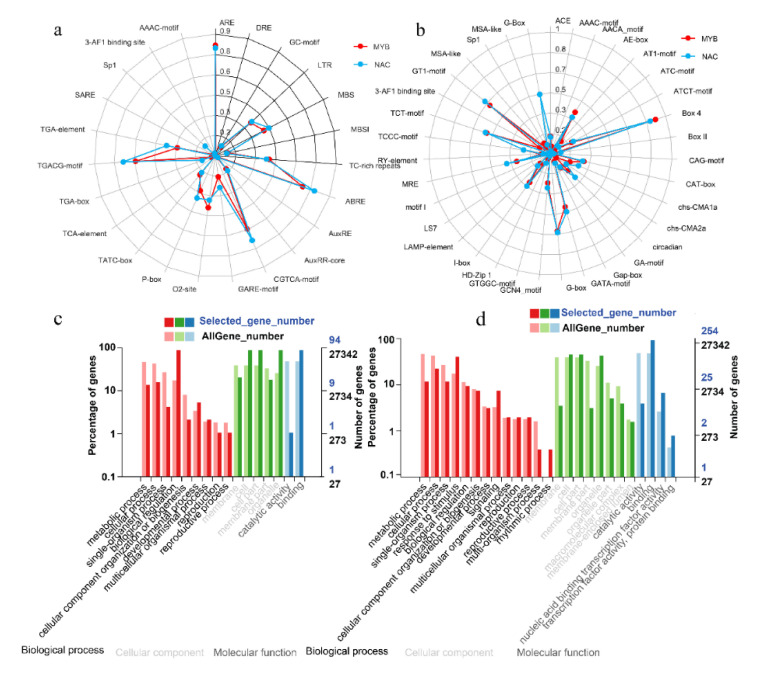
Statistics of cis-elements within the 2-kb promoter regions and functional classification of the *MaNAC* and *MaMYB* genes. (**a**) Stress- and hormone-responsive cis-elements within the promoter regions of the *MaNAC* and *MaMYB* genes. The blocks of black lines are stress-responsive cis-elements, while the blocks of gray lines are hormone-responsive cis-elements. (**b**) Development-responsive cis-elements within the promoter regions of the *MaNAC* and *MaMYB* genes. The red line represents *MaMYB*s, and the blue line represents *MaNAC*s. (**c**) *MaNAC* GO functional classification. (**d**) *MaMYB* GO functional classification.

The identification of cis-regulatory elements within promoter regions is beneficial for understanding the expression patterns of genes. The cis-elements in the promoter regions of *MaNAC*s and *MaMYB*s were similar and comprised stress-, hormone- and development-related cis-elements (Figure 5a,b). ARE cis-elements are essential for anaerobic induction [35]. ARE cis-elements were found in the promoter regions of 83.17% and 85.28% of the *MaNAC* and *MaMYB* genes, respectively (Figure 5a, Table S5). Low-temperature responsiveness (LTR) cis-elements were found in the promoter regions of 38.61% and 37.46% of the *MaNAC* and *MaMYB* genes, respectively (Figure 5a, Table S5). MBS and MBSI cis-elements are MYB-binding sites involved in drought inducibility and the regulation of flavonoid biosynthesis genes, respectively. In total, 46.53% of MaNAC and 42.14% of the MaMYB promoter regions had MBS cis-elements. MBSI cis-elements were found in 8.91% and 9.70% of the MaNAC and MaMYB gene promoter regions, respectively. Most development-related cis-elements are related to the light response, and the others were related to the endosperm, roots, seeds and the circadian rhythm (Figure 5b).

Given that NAC and MYB TFs play important roles in development and stress responses, Gene Ontology (GO) classification of MaNAC and MaMYB members was performed (Figure 5c,d, Table S6). The NAC family members were enriched mainly in biological regulation, developmental processes, and multicellular organismal processes (biological processes); cells, cell parts and organelles (cellular components); and binding (molecular functions) (Figure 5c). The MYB family members were enriched mainly in biological regulation, signaling and rhythmic process (biological process); cells, cell parts and organelles (cellular components); and binding, nucleic acid-binding TFs and TF activity, and protein binding (molecular functions) (Figure 5d).

### 2.6. Lignin Biosynthesis Genes Regulated by NAC Genes

**Figure 6:**
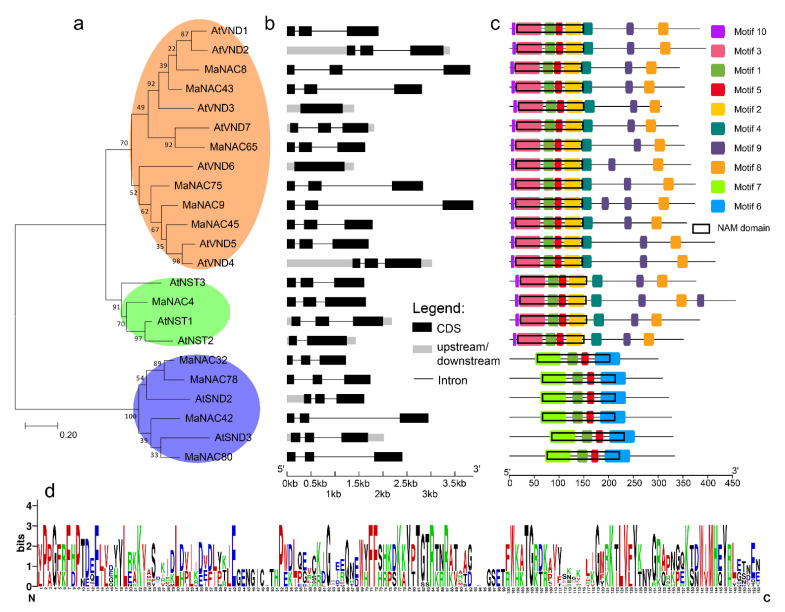
Phylogeny, gene structure and motif analysis of lignin biosynthesis-related NAC genes from *M. albus* and *Arabidopsis*. (**a**) Phylogenetic tree of 11 MaNAC proteins and 12 AtNAC proteins. The phylogenetic tree was constructed via MEGA 7.0, with the maximum likelihood method. The bootstrap test was performed with 1000 replicates. Three subgroups are shown in different colors. (**b**) Intron/exon structures of *M. albus* and *Arabidopsis NAC* genes. (**c**) *M. albus* and *Arabidopsis* NAC protein motifs and the position of the no apical meristem (NAM) domain. (**d**) Sequence logo of the NAM domain in *M. albus*. The bit score represents the information content for each position.

In the production and application of forage, lignin content significantly affects the quality of forage. NAC and MYB are known to be involved in regulating lignin biosynthesis. Using the Arabidopsis lignin-related NAC (AtVND1–7, AtNST1–3 and AtSND1-2) CDSs and protein sequences as queries, we identified six *VND* genes, one *NST* gene and four *SND* genes in *M. albus* (Figure 6a). We subsequently constructed a phylogenetic tree of *M. albus* and *Arabidopsis* NAC proteins according to the maximum likelihood method of MEGA 7.0 (Figure 6a). The 24 NAC members of *M. albus* and *Arabidopsis* were subdivided into three subgroups: the VND subgroup, NST subgroup and SND subgroup (Figure 6a). The similarity of amino acids was higher within the same subgroup than across subgroups (Figure S3). With the exceptions of AtVND3 and AtVND6, all these lignin biosynthesis-related genes had similar structures, with three exons (Figure 6b). Proteins in the VND and NST clades had the seven motifs, and proteins in the SND clade had four motifs (Figure 6c). All proteins had NAM domains that spanned motifs 1, 2, 3 and 5 in the VND and NST clades and motifs 1, 5, 6 and 7 in the SND clade (Figure 6c). The NAM domain with 153 amino acids was visualized by Gene Structures Display Server (GSDS) (Figure 6d). All amino acid sequences of *Arabidopsis* and *M. albus* were aligned by DNAMAN software; the results are shown in Figure S3.

### 2.7. Lignin Biosynthesis Genes Regulated by MYB Genes

**Figure 7:**
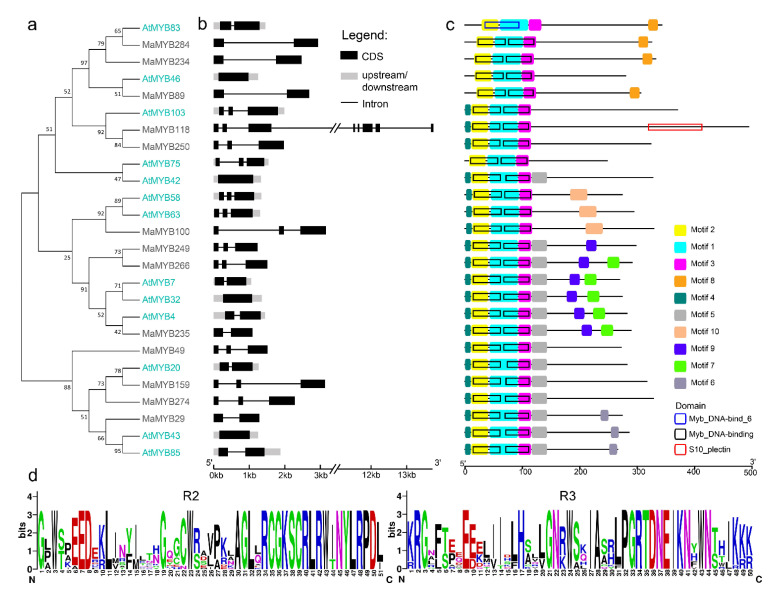
Phylogeny, gene structure and motif analysis of lignin biosynthesis-related *MYB* genes from *M. albus* and *Arabidopsis*. (**a**) Phylogenetic tree of 13 MaMYB proteins and 13 AtMYB proteins. The phylogenetic tree was constructed via MEGA, 7.0 with the maximum likelihood method. The bootstrap test was performed with 1000 replicates. (**b**) Intron/exon structures of *M. albus* and *Arabidopsis MYB* genes. (**c**) *M. albus* and *Arabidopsis* MYB protein motifs and the positions of the R2 and R3 Myb domains. (**d**) Sequence logo of the Myb domain in *M. albus*. The bit score represents the information content for each position.

Previous studies have shown that MYB members in *Arabidopsis* (MYB46, MYB83 [19], MYB20, MYB42, MYB43, MYB58, MYB63, MYB85 and MYB103 [22]) regulate lignin biosynthesis. The protein sequences and CDSs of these AtMYBs were used as queries to identify *M. albus* lignin biosynthesis-related *MaMYB* genes, and 13 *MaMYB*s were identified (Figure 7). On the basis of the AtMYB and MaMYB protein phylogenetic tree, two MYB83s (MaMYB234 and MaMYB284), one MYB46 (MaMYB89), two MYB103s (MaMYB118 and MaMYB250), one MYB58/MYB63 (MaMYB100), one MYB4 (MaMYB235), two MYB7/MYB32 (MaMYB266 and MaMYB249), two MYB43/MYB85 (MaMYB29 and MaMYB49), and two MYB20s (MaMYB159 and MaMYB274) were identified (Figure 7a). The gene structures of the *AtMYB*s and *MaMYB*s were similar, and the exon number varied from 1 to 3, all *MaMYB*s had more than two exons except for *MaMYB118* (Figure 7b). Except AtMYB83, all the MYB proteins contained motifs 1, 2 and 3 as well as two Myb DNA-binding domains, indicating that they are R2R3-MYBs (Figure 7c). The amino acid sequences of MYBs of *Arabidopsis* and *M. albus* were aligned by DNAMAN software; the results are shown in Figure S4. In addition, the R2 and R3 domains comprised 51 and 50 amino acids, respectively, and their structures were visualized by GSDS (Figure 7d).

### 2.8. NAC and MYB Gene Expression and Coexpression Analysis

**Figure 8:**
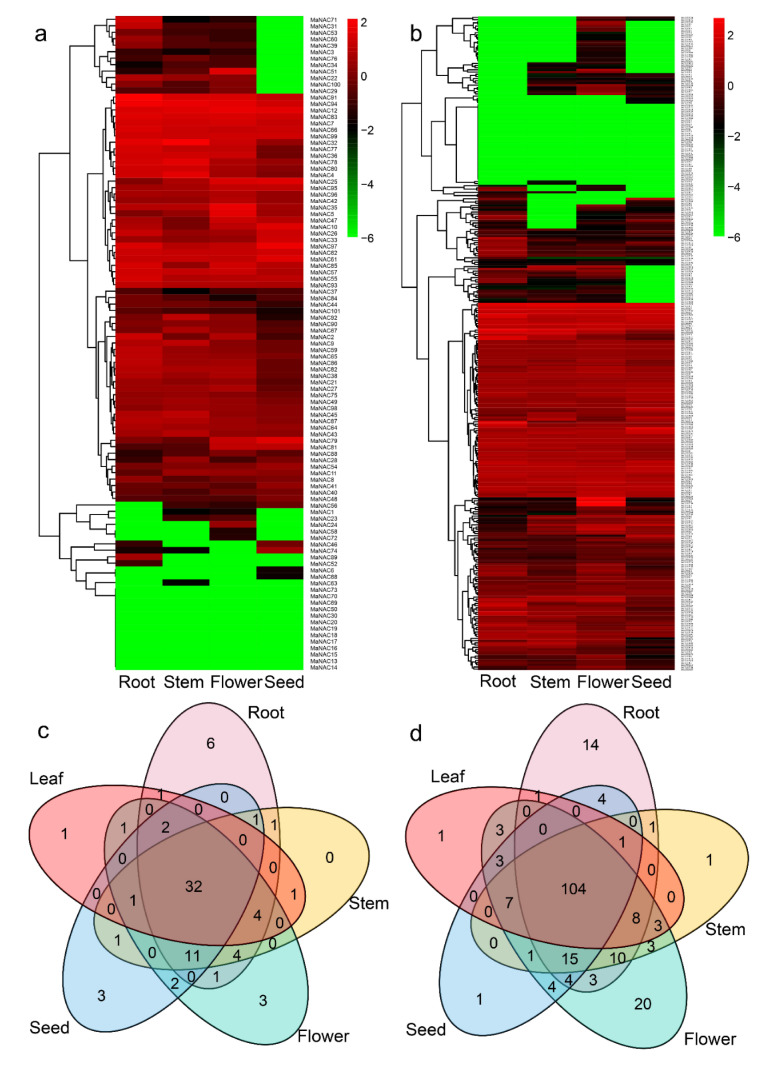
Expression profiles of *MaNAC*s and *MaMYB*s in the roots, stems, flowers and seeds. (**a**) Expression profiles of *MaNAC*s in the roots, stems, flowers and seeds. (**b**) Expression profiles of *MaMYB*s in the roots, stems, flowers and seeds. (**c**) The overlap of expressed *MaNAC* genes (FPKM > 0.5) in different tissues. (**d**) The overlap of expressed MaMYB genes (FPKM > 0.5) in different tissues.

To elucidate the possible roles of NACs and MYBs in the lignin biosynthesis, growth and development of *M. albus*, we analyzed RNA-seq data from different tissues. A total of 63 *MaNAC* genes and 185 *MaMYB*s were expressed in the roots, stems, flowers and seeds (Figure 8a,b, Tables S1 and S2). Some of the *MaNAC*s and *MaMYB*s were not expressed in any tissue, which indicated that they were functionally redundant. Thirty-two *MaNAC*s and 104 *MaMYB*s were expressed in all tissues. A total of 44 *MaNAC* genes and 108 *MaMYB*s presented tissue-specific expression (Figure 8c,d), among which 6 *MaNAC*s and 14 *MaMYB*s were only expressed in the roots, one *MaNAC* and one *MaMYB* were only expressed in the leaf, one *MaMYB* was only expressed in the stem, three *MaNAC*s and one *MaMYB* were only expressed in the seed, and three *MaNAC*s and 20 *MaMYB*s were only expressed in the flower. A total of 45 *MaNAC* genes and 128 *MaMYB* genes were expressed in five leaf samples (Figure S5). The expression level analysis suggested that tandem repeat genes exhibited similar expression patterns. For example, the expression levels of five *MaNAC* tandem repeat genes were notably low, and the expression patterns of three *MaNAC* tandem repeat genes were similar (Figure 8, Figure S5).

**Figure 9:**
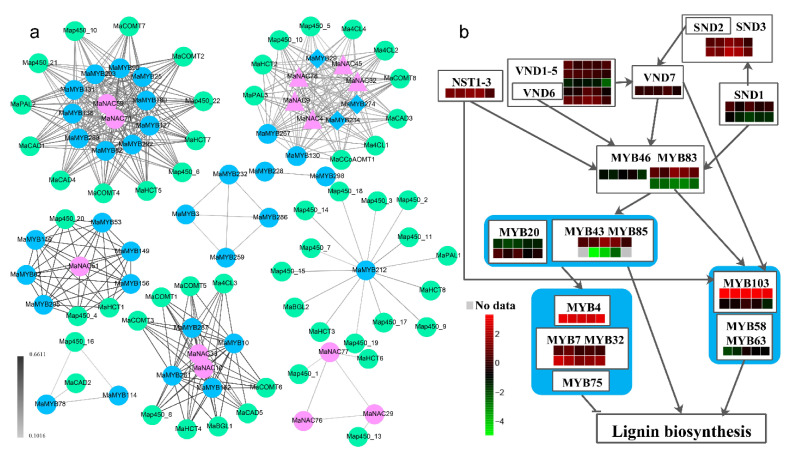
Coexpression network of *MaNAC*, *MaMYB* and lignin biosynthesis-related genes and their regulatory network. (**a**) Coexpression network of *MaNAC*, *MaMYB* and lignin biosynthesis-related genes. The green circle represents lignin biosynthesis-related genes that are expressed downstream of NACs and MYBs, the blue circle represents MYB TFs, the pink circle represents NAC TFs, the pink triangle represents lignin biosynthesis-related NAC genes, and the blue rhombus represents lignin biosynthesis-related MYB genes. 4CL: 4-coumarate CoA Ligase, BGL: β-glucosidase, CAD: cinnamyl alcohol dehydrogenase, CCoAOMT: caffeoyl CoA O-methyltransferase, COMT: caffeic acid O-methyltransferase, HCT: hydroxycinnamoyl transferase, PAL: phenylalanine ammonia-lyase. The color of the line represents the weight value between two nodes. (**b**) Regulatory network of *MaNAC*, *MaMYB* and lignin biosynthesis-related genes. The heatmap below the gene name shows the gene expression pattern of five leaf samples. The heatmap represents the gene number in the horizontal direction and the expression level in the vertical direction for five leaf samples.

Several NAC and MYB family members are involved in lignin biosynthesis by regulating lignin biosynthesis genes (*PAL1*, *C4H*, *4CL1*, *CCoAOMT*, *HCT*, *CCR1*, and *F5H1*) [36,37,38,39]. To determine the relationship of MaNAC and MaMYB TFs further, we performed a weighted gene coexpression network analysis (WGCNA) based on RNA-seq data of the roots, stems, flowers, and seeds as well as five leaf samples, with three replications. Thirteen *MaNAC*s, 34 *MaMYB*s, and 53 lignin biosynthesis-related genes were identified (Table S7), which exhibited a coexpression relationship (Figure 9a). The coexpression network comprised 499 pairs, and each pair of nodes was directly or indirectly connected to each component (Figure 9a). MaNAC and MaNAC members, MaMYB and MaMYB members, MaNAC and MaMYB members, MaNAC and lignin biosynthesis genes, and MaMYB and lignin biosynthesis genes presented coexpression relationships, albeit with different weight values (Figure 9a). In particular, MaNAC4 (NST), MaNAC9 (VND), MaNAC32 (SND), MaNAC45 (VND6), MaNAC78 (SND), MaMYB29 (MYB43/85), MaMYB234 (MYB46/83) and MaMYB274 (MYB20/42) presented coexpression relationships with each other, and all of them presented coexpression relationships with 10 lignin biosynthesis genes (Figure 9a).

### 2.9. Network of Lignin Biosynthesis-Related MaNACs and MaMYBs

**Figure 10:**
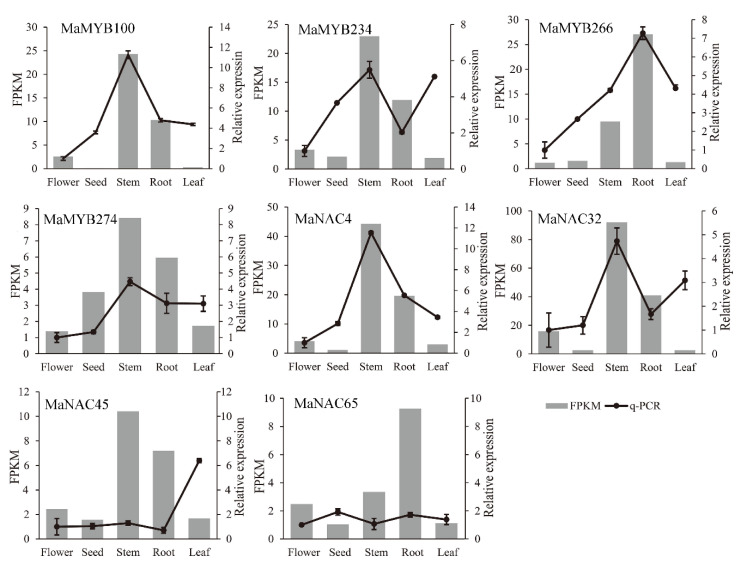
Expression of NAC and MYB lignin biosynthesis-related genes determined by qRT-PCR in flower, seed, stem, root and leaf. The shown values are the means ± standard deviation of three replicates. *MaTublin* was used as the reference gene.

The first layer of master switch genes (SND1/NST3, NST1, VND6, and VND7) and the second layer of master switch genes (MYB46 and MYB83) regulate downstream genes to control lignin biosynthesis during secondary wall formation [19]. MYB46 and MYB83 are direct targets of NST3/SND1, VND6 and VND7 [20,40,41,42]. MYB103 is a direct target of NST1, which is involved in syringyl lignin biosynthesis [43] and may be a direct target of VND6 and VND7 [41,42]. *AtMYB58*, *AtMYB63*, and *AtMYB103* are direct targets of *AtMYB46/83* [22]. *AtMYB20*, *AtMYB42*, *AtMYB43*, and *AtMYB85* activate *AtMYB4*, *AtMYB7*, and *AtMYB32* and directly activate lignin biosynthesis genes during secondary wall formation in *Arabidopsis* [22]. *AtMYB58*, *AtMYB63*, and *AtMYB103* regulate lignin biosynthesis genes [19,21,44], and *AtMYB4*, *AtMYB32*, and *AtMYB7* negatively regulate lignin biosynthesis genes [19]. On the basis of previous studies, we assumed a *MaNAC* and *MaMYB* coregulation network (Figure 9b). The expression pattern of *MaMYB234* was opposite that of *MaNAC43* and *MaNAC8* (VND) and *MaMYB29*. The expression pattern of *MaMYB234* was similar to that of *MaNAC78* and *MaNAC32*, *MaMYB118*, *MaMYB235* and *MaMYB249* and *MaMYB266* (Figure 9b). In the different examined tissues, most of the genes (16/24) were highly expressed in the stems. However, *MaMYB118*, *MaMYB266*, *MaNAC8* and *MaNAC65* (VND) were highly expressed in the roots. To further validate the reliability of gene expression data obtained by RNA-Seq analysis, four lignin biosynthesis-related NAC and MYB genes each carried out qRT–PCR analysis in five tissues (Figure 10). NAC and MYB genes were highly expressed in stems and roots, which were consistent with the RNA-seq analysis, with the exception of three genes higher expressed in leaf than in stem or root.

## 3. Discussion

NACs and MYBs compose two large TF families. As an increasing number of genomes of legume species are sequenced, additional NAC and MYB TF family members are being identified. For instance, there are, respectively, 269 and 438 NACs and MYBs in *Glycine max*, 123 and 185 in *M. truncatula*, 116 and 126 in *Lotus japonicus*, and 96 and 166 in *Cicer arietinum* (data from PlantTFDB 5.0, http://planttfdb.cbi.pku.edu.cn/index.php?sp=Mtr, accessed on 28 February 2020). In the present study, based on whole-genome data of the *M. albus*, 101 *MaNAC* and 299 *MaMYB* genes were identified. Because *G. max* underwent independent whole-genome duplication, there are substantially more of these family members in this species than other legume species. The number of NACs is similar between *M. albus* and both *L. japonicus* and *C. arietinum*, but there are substantially more MYBs in *M. albus* than in *M. truncatula*, *L. japonicus* and *C. arietinum*. *M. albus* can adapt to drought, cold and high-salinity environments [45], and the expansive MaMYB family may contribute to the increased stress tolerance of *M. albus*. The phylogenetic tree *M. albus*, *M. truncatula*, rice and *Arabidopsis* showed that most clades contained NAC or MYB members of all three species, but some clades contained members of only one species. In addition, the intron/exon and motif structures of NAC and MYB genes were similar in one clade. Genes in the same clade may share the same ancestor before species divergence, and genes in independent clades with genes from only one species may have formed after species divergence.

Previous studies have shown that NAC TFs regulate cell division [46] and flowering in response to salt stress [47] and are involved in the regulation of the drought response [1] and in AtNAC1-mediated auxin signaling to promote lateral root development [48]. In addition, the various functions of MYB TFs in plants include lignin biosynthesis [49,50], hormone signal transduction [51,52], stress tolerance [53,54], and organ development [55,56]. The promoter regions of *MaNAC*s and *MaMYB*s contain stress-, development- and hormone-responsive cis-elements. Some of the gene promoter regions contain ARE cis-elements (>80%) and LTR cis-elements (>37%), which are involved in stress responses [35]. CAT-box cis-elements are related to meristem expression, GCN4_motif cis-elements are involved in endosperm expression, CGTCA-motif cis-elements are involved in methyl jasmonate (MeJA) responsiveness, and TCA elements are involved in salicylic acid responsiveness [35]. The various cis-elements within the promoter regions of *MaNAC*s and *MaMYB*s are strongly associated with gene function. Furthermore, GO annotation of the *MaNAC*s and *MaMYB*s indicated that they are associated with stimulus response and development, and tissue-specific expression of the *MaNAC* and *MaMYB* genes indicated that these genes may regulate organ development. Furthermore, 23 *MaMYB* genes were expressed only in the flowers, suggesting that they may regulate *M. albus* floral development. Therefore, MaNAC and MaMYB members may be involved in stress, development and hormone responses.

Some NAC members target MYB members that are involved in lignin biosynthesis [19]. Analysis of the cis-elements within the promoter regions revealed that the promoters of some *MaNAC*s and *MaMYB*s contained MBS and MBSI cis-elements. Moreover, WGCNA indicated that *MaNAC* and *MaMYB* members presented coexpression relationships. Our results are consistent with those of previous studies, which showed that NAC members regulate MYB members. On the basis of the relationship between MYBs and NACs, and MYBs in *Arabidopsis*, a network comprising *MaNAC*s and *MaMYB*s with respect to lignin biosynthesis in *M. albus* was constructed which further confirmed the *MaNAC* and *MaMYB* relationship. Stems and roots are highly lignified, and our sequencing results are consistent with this fact. Comparing gene expression levels in different tissues, we identified genes that are highly expressed in the stems and roots, as well as flower-specific genes. Furthermore, the results of qRT-PCR also showed higher relative expression level in stems and roots. VND and NST subfamily members may function redundantly [19], compared with seven AtVNDs and three AtNSTs in *Arabidopsis*, only six VNDs and one NST were identified in *M. albus*. Two SND2s, two SND3s, two MYB83s, two MYB20s, and two MYB103s were identified that had expanded in *M. albus* compared with *Arabidopsis*. Except for *MaMYB284*, all these genes were expressed in different tissues. These expanded genes may increase *M. albus* lignin biosynthesis, as mature *M. albus* plants have a greater degree of lignification than mature *Arabidopsis* plants.

## 4. Materials and Methods

### 4.1. Genome-Wide Identification of NAC and MYB Genes in M. albus

The protein sequences of *Arabidopsis*, *Oryza sativa* and *Medicago truncatula* NAC and MYB TFs were downloaded from the Plant Transcription Factor Database (http://planttfdb.cbi.pku.edu.cn/index.php, accessed on 24 February 2020). Similarly, gene sequences and coding sequences (CDSs) were downloaded from the Phytozome 12 (https://phytozome.jgi.doe.gov/pz/portal.html#, accessed on 24 February 2020) database (Table S8). Local BLASTP and BLASTN searches were performed to identify the candidate NAC and MYB members, with *Arabidopsis* and *O. sativa* CDS and protein sequences used as queries; an e-value cut-off of 1e^−5^ was applied for homolog recognition. To confirm the candidate NAC and MYB genes, protein sequences were submitted to the Pfam website (http://pfam.xfam.org/search/keyword?query=&submit=Submit#tabview=tab1, accessed on 20 March 2020) with default parameters. NAC candidate sequences without an NAM domain and MYB candidate sequences without a Myb domain were discarded manually. Gene, coding, and protein sequences of NAC and MYB family members are deposited in NCBI.

Sequences of the *Arabidopsis* lignin biosynthesis-related NAC and MYB genes were retrieved from previous studies and used as queries to identify the corresponding *M. albus* genes. Local BLAST searches were performed: an e-value cut-off of 1e^−100^ and 1e^−70^ was applied to NAC and MYB homologous sequence recognition, respectively (Table S9).

### 4.2. Chromosomal Location, Motif Analysis, and Gene Structure of the NAC and MYB Genes in M. albus

Each *MaNAC* and *MaMYB* gene was mapped to the *M. albus* genome to determine its position on the chromosome via TBtools with default parameters [57]. The protein sequences of the NAC and MYB family members were submitted to MEME (http://meme-suite.org/tools/meme, accessed on 25 March 2020) (with the default parameters) to identify conserved motifs. To examine the conserved domains of *M. albus* in detail, multiple amino acid sequence alignment was performed via DNAMAN 6.0 software. The multiple alignment sequences were submitted to WebLogo (http://weblogo.berkeley.edu/logo.cgi, accessed on 3 April 2020) to create sequence logos of NAM and R2 and R3 Myb repeats. Furthermore, the CDSs of NAC and MYB were compared with their corresponding genomic sequences to determine all gene structures, which were visualized using the Gene Structure Display Server (http://gsds.cbi.pku.edu.cn/, accessed on 10 April 2020) with default parameters.

### 4.3. Phylogenetic Analysis

The NAC and MYB protein sequences of *Arabidopsis*, *O. sativa* and *M. albus* were aligned via the Muscle method, which is the MEGA 7.0 built-in method. Phylogenetic trees of NAC and MYB family members were then constructed by MEGA 7.0 [58] with the neighbor-joining (NJ) method (bootstraps with 1000 replicates). Lignin biosynthesis-related NAC and MYB phylogenetic trees were subsequently constructed according to the maximum likelihood method.

### 4.4. Analysis of the Promoter Regions of MaNAC and MaMYB Genes

The 2000-bp upstream of the transcriptional start site of *MaNAC* and *MaMYB* genes was extracted from the *M. albus* genome to identify the cis-elements within the promoter regions. The plant Cis-Acting Regulatory Element (http://bioinformatics.psb.ugent.be/webtools/plantcare/html/, accessed on 9 May 2020) database was used to identify cis-elements within the promoter regions of each gene (with the default parameters), and the function of each cis-element was annotated.

### 4.5. Synteny Analysis of MYB and NAC Genes

Genes within families were aligned with one another by the use of BLAST (e-value < 1 × 10^−10^), and the best matched gene pairs were defined as homologous pairs. The synonymous substitution rates (Ks) and nonsynonymous substitution rates (Ka) of the gene pairs were calculated by TBtools [57].

The syntenic relationships of *M. albus* genes were determined via MCScanX [59], with the default parameters. The MYB and NAC gene pairs were then selected from the MCScanX results. Circos [60] was subsequently used to visualize the syntenic relationships.

### 4.6. Expression Analysis and Gene Annotation

Seeds were grown in turfy soil in a glasshouse, which was maintained at 28/24 °C (day/night) and provided a 16 h light/8 h dark photoperiod. During the inflorescence stage, tissue samples of fresh stems, flowers and roots were collected, immediately frozen in liquid nitrogen and then stored at −80 °C. Seed samples were mixed together, imbibed and then allowed to germinate for 2 days. Each sample was a composite of independent biological replicates.

Total RNA from each sample was isolated by the use of TRIzol reagent (Invitrogen) according to the manufacturer’s instructions. Messenger RNAs (mRNAs) were separated from the total RNA by oligo (dT) primers and were cleaved into short fragments at random. Sequencing libraries were generated via rRNA-depleted RNA with a NEBNext^®^ Ultra™ Directional RNA Library Prep Kit for Illumina^®^ (NEBNext, Ipswich, MA, USA) according to the manufacturer’s recommendations. The qualified cDNA libraries were constructed by PCR enrichment and sequenced on a HiSeq 2500 platform, with a read length of 125 bp. Three independent biological replicates were sequenced per sample. The RNA sequencing (RNA-seq) data of leaf samples from 5 different genotypes from a previous study were also included [32].

Clean reads were obtained by removing reads containing adapters, reads containing poly-N sequences and reads of low quality from the raw reads. The clean reads were mapped to the *M. albus* genome by HISAT2. StringTie 1.3.1 [61] was used to calculate fragments per kilobase of transcript per million mapped reads (FPKMs) of mRNAs in each sample. The FPKMs of the genes were computed by summing the FPKMs of the transcripts in each gene group. Genes with FPKM < 0.5 were defined as non-expressed genes.

All gene expression levels were used to calculate the weight of gene pairs via WGCNA. The relationships of the MYB and NAC gene pairs were visualized by Cytoscape 3.5.1, and gene functions were annotated via the Gene Ontology (GO) database.

PrimeScript^®^ RT reagent Kit (TaKaRa, Kyoto, Japan) was used to reverse transcribe RNA into first-stand cDNA. The product was used as a template for qRT-PCR with specific primers (Table S10) which were designed by PerlPrimer v1.1.21. MaTublin was used as the reference gene. qRT-PCR was performed using SYBR Premix Ex TaqTM (TaKaRa). Each experiment was performed with three replicates. The relative transcription levels were calculated by the comparative delta-delta Ct method.

## 5. Conclusions

In summary, a total of 101 MaNAC and 299 MaMYB family members were identified in *M. albus*. Phylogenetic and evolutionary relationships, gene structure analysis and motif analysis indicated the conservation of MaNAC and MaMYB families and that some members diverged from their ancestors. MaNAC and MaMYB members experienced purifying selection and some of the members were functionally redundant. Promoter region analysis and GO functional annotation of MaNAC and MaMYB TFs suggested that they may be involved in stress, development and hormone responses. WGCNA revealed that *MaNAC*, *MaMYB* and lignin biosynthesis-related genes presented coexpression relationships, and a regulatory network comprising some of these genes in *M. albus* further confirmed their relationship. Gene expression levels in different tissues were compared, some of the MaNAC and MaMYB members presented tissue-specific expression, lignin biosynthesis-related MaNACs and MaMYBs were highly expressed in stem and root, and the relative expression of several members was also high in stem and root, which further confirmed that these candidate genes may regulated lignin biosynthesis. These results provide a useful framework for future research to understand the evolution and function of the MaNAC and MaMYB gene families and to understand the potential physiological role of the *MaNAC* and *MaMYB* genes during lignin biosynthesis. Since no *MaNAC* and *MaMYB* genes have been functionally characterized to date, our results may facilitate future efforts to breed forage cultivars with low amounts of lignin.

## Data Availability

The data presented in this study are openly available: *M. albus* genome data are available from NCBI, with BioProject ID: PRJNA674670. The raw transcriptome sequences of tissues have been deposited in the SRA of NCBI with BioProject ID: PRJNA647665. The gene and protein sequences of MaMYB and MaNAC have been deposited in NCBI with GenBank accession numbers: MW302361—MW302760.

## Supplementary Materials

The following are available online at https://www.mdpi.com/2223-7747/10/2/303/s1, Figure S1: Intron/exon structure of MaMYBs, Figure S2: Motif structure of MaMYBs, Figure S3: Alignment of lignin biosynthesis-related MaNACs amin acids using DNAMAN software, Figure S4: Alignment of lignin biosynthesis-related MaMYBs amin acids using DNAMAN software, Figure S5: Expression profiles of *MaNAC*s and *MaMYB*s in the roots, stems, flowers, and seeds. Table S1: The MaMYB family member information and FPKM values, and myb domain type of MaMYB, Table S2: The MaNAC family member information and FPKM values, Table S3: Synteny gene pairs of NAC and MYB family members, Table S4: The value of Ka, Ks, and Ka/Ks of MaNAC or MaMYB gene pairs, Table S5: cis-element types in the promoter region of MaNAC or MaMYB genes, Table S6: GO classification analysis of NAC (a) and MYB (b) gene family, Table S7: Lignin biosynthesis-related genes information, Table S8: Query gene sequences, a: OsMYB protein sequences, b: OsMYB coding sequences, c: AtMYB protein sequences, d: AtMYB coding sequences, e: MtMYB protein sequences, f: OsNAC protein sequences, g: OsNAC coding sequences, h: AtNAC protein sequences, i: AtNAC coding sequences, j: MtNAC protein sequences, Table S9: Homologous genes of NAC (a) and MYB (b) family among Melilotus albus, Medicago truncatula, Arabidopsis thaliana and Orzya sativa, Table S10: qRT-PCR primers of selected genes.

## References

[1] Le D.T., Nishiyama R., Watanabe Y., Mochida K., Yamaguchi-Shinozaki K., Shinozaki K., Tran L.S. (2011). Genome-wide survey and expression analysis of the plant-specific NAC transcription factor family in soybean during development and dehydration stress. DNA Res..

[2] Tongkun L., Xiaoming S., Weike D., Zhinan H., Gaofeng L., Ying L., Xilin H. (2014). Genome-Wide Analysis and Expression Patterns of NAC Transcription Factor Family Under Different Developmental Stages and Abiotic Stresses in Chinese Cabbage. Plant Mol. Biol. Rep..

[3] Ooka H., Satoh K., Doi K., Nagata T., Otomo Y., Murakami K., Matsubara K., Osato N., Kawai J., Carninci P. (2003). Comprehensive analysis of NAC Family genes in *Oryza sativa* and *Arabidopsis thaliana*. DNA Res..

[4] Kim S.M., Kim S.-G., Kim Y.-S., Seo P.J. (2007). Exploring membrane-associated NAC transcription factors in Arabidopsis: Implications for membrane biology in genome regulation. Nucleic Acids Res..

[5] Kim S.G., Lee S., Seo P.J., Kim S.K., Kim J.K., Park C.M. (2010). Genome-scale screening and molecular characterization of membrane-bound transcription factors in Arabidopsis and rice. Genomics.

[6] Hu H., Dai M., Yao J., Xiao B., Xiong L. (2006). Overexpressing a NAM, ATAF, and CUC (NAC) transcription factor enhances drought resistance and salt tolerance in rice. Proc. Natl. Acad. Sci. USA.

[7] Jensen M.K., Hagedorn P.H., Torres-Zabala M.D., Grant M.R., Lyngkjaer M.F. (2008). Transcriptional regulation by an NAC (NAM-ATAF1,2-CUC2) transcription factor attenuates ABA signalling for efficient basal defence towards Blumeria graminis f. sp. hordei in Arabidopsis. Plant J..

[8] Dubos C., Stracke R., Grotewold E., Weisshaar B., Martin C., Lepiniec L. (2010). MYB transcription factors in Arabidopsis. Trends Plant Sci..

[9] Ogata K., Kanei-Ishii C., Sasaki M., Hatanaka H., Nagadoi A., Enari M., Nakamura H., Nishimura Y., Ishii S., Sarai A. (1996). The cavity in the hydrophobic core of Myb DNA-binding domain is reserved for DNA recognition and trans-activation. Nat. Struct. Mol. Biol..

[10] Kirik V. (2005). Functional diversification of MYB23 and GL1 genes in trichome morphogenesis and initiation. Development.

[11] Li S.F., Milliken O.N., Pham H., Seyit R., Napoli R., Preston J., Koltunow A.M., Parish R.W. (2009). The Arabidopsis MYB5 Transcription Factor Regulates Mucilage Synthesis, Seed Coat Development, and Trichome Morphogenesis. Plant Cell.

[12] Millar A.A. (2005). The *Arabidopsis* GAMYB-like genes, MYB33 and MYB65, are microRNA-regulated genes that redundantly facilitate anther development. Plant Cell.

[13] Seo P.J., Xiang F., Qiao M., Park J.Y., Park C.M. (2009). The MYB96 Transcription Factor Mediates Abscisic Acid Signaling during Drought Stress Response in Arabidopsis. Plant Physiol..

[14] Qiao Z. (2016). Lignification: Flexibility, Biosynthesis and Regulation. Trends Plant Sci..

[15] Weng J.K., Chapple C. (2010). The origin and evolution of lignin biosynthesis. New Phytol..

[16] Ohtani M., Demura T. (2018). The quest for transcriptional hubs of lignin biosynthesis: Beyond the NAC-MYB-gene regulatory network model. Curr. Opin. Biotechnol..

[17] Minoru K., Makiko U., Nobuyuki N., Gorou H., Masatoshi Y., Jun I., Tetsuro M., Hiroo F., Taku D. (2005). Transcription switches for protoxylem and metaxylem vessel formation. Genes Dev..

[18] Mitsuda N., Iwase A., Yamamoto H., Yoshida M., Seki M., Shinozaki K., Ohme-Takagi M. (2007). NAC Transcription Factors, NST1 and NST3, Are Key Regulators of the Formation of Secondary Walls in Woody Tissues of Arabidopsis. Plant Cell.

[19] Nakano Y., Yamaguchi M., Endo H., Rejab N.A., Ohtani M. (2015). NAC-MYB-based transcriptional regulation of secondary cell wall biosynthesis in land plants. Front. Plant Sci..

[20] Ko J.H., Jeon H.W., Kim W.C., Kim J.Y., Han K.H. (2014). The MYB46/MYB83-mediated transcriptional regulatory programme is a gatekeeper of secondary wall biosynthesis. Ann. Bot..

[21] Zhou J., Lee C., Zhong R., Ye Z.H. (2009). MYB58 and MYB63 are transcriptional activators of the lignin biosynthetic pathway during secondary cell wall formation in Arabidopsis. Plant Cell.

[22] Pan G., Su Z., Jinyue L., Cuihuan Z., Jie W., Yingping C., Chunxiang F., Xue H., Hang H., Qiao Z. (2020). MYB20, MYB42, MYB43, and MYB85 regulate phenylalanine and lignin biosynthesis during secondary cell wall formation. Plant Physiol..

[23] Smith W.K., Gorz H.J. (1965). Sweetclover improvement. Adv. Agron..

[24] Wunderlin R. (1982). The Leguminosae: A source book of characteristics, uses, and nodulation. Econ. Bot..

[25] Clark A. (2007). Managing Cover Crops Profitably.

[26] Jung H.G., Allen M.S. (1995). Characteristics of plant cell walls affecting intake and digestibility of forages by ruminants. J. Anim. Sci..

[27] Wu F., Zhang D., Ma J., Luo K., Di H., Liu Z., Zhang J., Wang Y. (2016). Analysis of genetic diversity and population structure in accessions of the genus *Melilotus*. Ind. Crop. Prod..

[28] Di H., Duan Z., Luo K., Zhang D., Wu F., Zhang J., Liu W., Wang Y. (2015). Interspecific Phylogenic Relationships within Genus Melilotus Based on Nuclear and Chloroplast DNA. PLoS ONE.

[29] Zhang J., Hongyan D., Kai L., Zulfi J., Fan W., Zhen D., Alan S., Zhuanzhuan Y., Yanrong W. (2018). Coumarin content, morphological variation, and molecular phylogenetics of *Melilotus*. Molecules.

[30] Wu F., Ma J., Meng Y., Zhang D., Pascal Muvunyi B., Luo K., Di H., Guo W., Wang Y., Feng B. (2017). Potential DNA barcodes for Melilotus species based on five single loci and their combinations. PLoS ONE.

[31] Luo K., Jahufer M.Z.Z., Zhao H., Zhang R., Wu F., Yan Z., Zhang J., Wang Y. (2017). Genetic improvement of key agronomic traits in *Melilotus albus*. Crop Sci..

[32] Wu F., Luo K., Yan Z., Zhang D., Yan Q., Zhang Y., Yi X., Zhang J. (2018). Analysis of miRNAs and their target genes in five Melilotus albus NILs with different coumarin content. Sci. Rep..

[33] Luo K., Wu F., Zhang D., Dong R., Fan Z., Zhang R., Yan Z., Wang Y., Zhang J. (2017). Transcriptomic profiling of Melilotus albus near-isogenic lines contrasting for coumarin content. Sci. Rep..

[34] Jiang X., Assis R. (2019). Rapid functional divergence after small-scale gene duplication in grasses. BMC Evol. Biol..

[35] Shariatipour N., Heidari B. (2020). Meta-analysis of expression of the stress tolerance associated genes and uncover their cis-regulatory elements in rice (*Oryza sativa* L.). Open Bioinform. J..

[36] Kim W., Kim J., Ko J., Kang H., Han K. (2014). Identification of direct targets of transcription factor MYB46 provides insights into the transcriptional regulation of secondary wall biosynthesis. Plant Mol. Biol..

[37] Kim W.C., Kim J., Ko J., Kim J., Han K. (2013). Transcription factor MYB46 is an obligate component of the transcriptional regulatory complex for functional expression of secondary wall-associated cellulose synthases in *Arabidopsis thaliana*. J. Plant Physiol..

[38] Kim W.C., Ko J.H., Han K.H. (2012). Identification of acis-acting regulatory motif recognized by MYB46, a master transcriptional regulator of secondary wall biosynthesis. Plant Mol. Biol..

[39] Kim W.C., Ko J.H., Kim J.Y., Kim J., Bae H.J., Han K.H. (2013). MYB46 directly regulates the gene expression of secondary wall-associated cellulose synthases in *Arabidopsis*. Plant J..

[40] McCarthy R.L., Zhong R., Ye Z.H. (2009). MYB83 is a direct target of SND1 and acts redundantly with MYB46 in the regulation of secondary cell wall biosynthesis in Arabidopsis. Plant Cell Physiol..

[41] Kyoko O.I., Yoshihisa O., Hiroo F. (2010). *Arabidopsis* VASCULAR-RELATED NAC-DOMAIN6 directly regulates the genes that govern programmed cell death and secondary wall formation during xylem differentiation. Plant Cell.

[42] Yamaguchi M., Mitsuda N., Ohtani M., Ohme-Takagi M., Demura T. (2011). VASCULAR-RELATED NAC-DOMAIN 7 directly regulates the expression of a broad range of genes for xylem vessel formation. Plant J..

[43] Ohman D., Demedts B., Kumar M., Gerber L., Sundberg B. (2013). MYB103 is required for FERULATE-5-HYDROXYLASE expression and syringyl lignin biosynthesis in Arabidopsis stems. Plant J. Cell Mol. Biol..

[44] Zhong R., Lee C., Zhou J., Mccarthy R.L., Ye Z.H. (2008). A Battery of Transcription Factors Involved in the Regulation of Secondary Cell Wall Biosynthesis in Arabidopsis. Plant Cell.

[45] Rogers M.E., Colmer T.D., Frost K., Henry D., Cornwall D., Hulm E., Deretic J., Hughes S.R., Craig A.D. (2008). Diversity in the genus *Melilotus* for tolerance to salinity and waterlogging. Plant Soil.

[46] Kim Y.S., Kim S.G., Park J.E., Park H.Y., Lim M.H., Chua N.H., Park C.M. (2006). A membrane-bound NAC transcription factor regulates cell division in *Arabidopsis*. Plant Cell.

[47] Kim S.G., Kim S.Y., Park C.M. (2007). A membrane-associated NAC transcription factor regulates salt-responsive flowering via FLOWERING LOCUS T in *Arabidopsis*. Planta.

[48] Xie Q. (2000). *Arabidopsis* NAC1 transduces auxin signal downstream of TIR1 to promote lateral root development. Genes Dev..

[49] Goicoechea M., Lacombe E., Legay S., Mihaljevic S., Grima-Pettenati J. (2005). EgMYB2, a new transcriptional activator from Eucalyptus xylem, regulates secondary cell wall formation and lignin biosynthesis. Plant J..

[50] Bedon F., Jacqueline G.P., John M. (2007). Conifer R2R3-MYB transcription factors: Sequence analyses and gene expression in wood-forming tissues of white spruce (*Picea glauca*). BMC Plant Biol..

[51] Abe H. (2002). *Arabidopsis* AtMYC2 (bHLH) and AtMYB2 (MYB) Function as Transcriptional Activators in Abscisic Acid Signaling. Plant Cell.

[52] Yeon K., Xiaohua L., Sun Ju K., Haeng K., Jeongyeo L., Hyeran K., Sang P. (2013). MYB transcription factors regulate glucosinolate biosynthesis in different rrgans of Chinese cabbage (*Brassica rapa* ssp. pekinensis). Molecules.

[53] Hartmann U., Sagasser M., Mehrtens F., Stracke R., Weisshaar B. (2005). Differential combinatorial interactions of cis-acting elements recognized by R2R3-MYB, BZIP, and BHLH factors control light-responsive and tissue-specific activation of phenylpropanoid biosynthesis genes. Plant Mol. Biol..

[54] Jung C., Seo J.S., Han S.W., Koo Y.J., Kim C.H., Song S.I., Nahm B.H., Choi Y.D., Cheong J.-J. (2007). Overexpression of AtMYB44 enhances stomatal closure to confer abiotic stress tolerance in transgenic *Arabidopsis*. Plant Physiol..

[55] Maria P.R., Felix J., Eugenio B., Cathie M. (2005). Development of three different cell types is associated with the activity of a specific MYB transcription factor in the ventral petal of *Antirrhinum majus* flowers. Development.

[56] Baumann K., Perez Rodriguez M., Bradley D., Venail J., Martin C. (2007). Control of cell and petal morphogenesis by R2R3 MYB transcription factors. Development.

[57] Chen C., Xia R., Chen H., He Y. (2018). TBtools, a Toolkit for Biologists integrating various HTS-data handling tools with a user-friendly interface. bioRxiv.

[58] Kumar S., Stecher G., Tamura K. (2016). MEGA7: Molecular Evolutionary Genetics Analysis Version 7.0 for Bigger Datasets. Mol. Biol. Evol..

[59] Wang Y., Tang H., Debarry J.D., Tan X., Li J., Wang X., Lee T.-H., Jin H., Marler B., Guo H. (2012). MCScanX: A toolkit for detection and evolutionary analysis of gene synteny and collinearity. Nucleic Acids Res..

[60] Krzywinski M., Schein J., Birol I. (2009). Circos: An information aesthetic for comparative genomics. Genome Res..

[61] Mihaela P., Pertea G.M., Antonescu C.M., Tsung-Cheng C., Mendell J.T., Salzberg S.L. (2015). StringTie enables improved reconstruction of a transcriptome from RNA-seq reads. Nat. Biotechnol..

