# Supplementary material for: NAC and MYB Families and Lignin Biosynthesis-Related Members Identification and Expression Analysis in Melilotus albus
Source: Plants (Basel). 2021 Feb 5;10(2):303. doi: 10.3390/plants10020303 (PMC7914948; doi:10.3390/plants10020303)
Supplement: Supplementary file 1 [file plants-10-00303-s001.zip › plants-956005-supp-final/Supplementary Files/Supplementary Figures.docx]

*Article*

**NAC and MYB Families and Lignin Biosynthesis-Related Members Identification and Expression Analysis in *Melilotus Albus***

**Lijun Chen, Fan Wu and Jiyu Zhang ***

State Key Laboratory of Grassland Agro-ecosystems; Key Laboratory of Grassland Livestock Industry Innovation, Ministry of Agriculture and Rural Affairs; Engineering Research Center of Grassland Industry, Ministry of Education; College of Pastoral Agriculture Science and Technology, Lanzhou University, Lanzhou 730020, China; Lijun Chen, chenlj16@lzu.edu.cn (L.C.); Fan Wu, wuf15@lzu.edu.cn (F.W.)

***** Correspondence: Jiyu Zhang, zhangjy@lzu.edu.cn


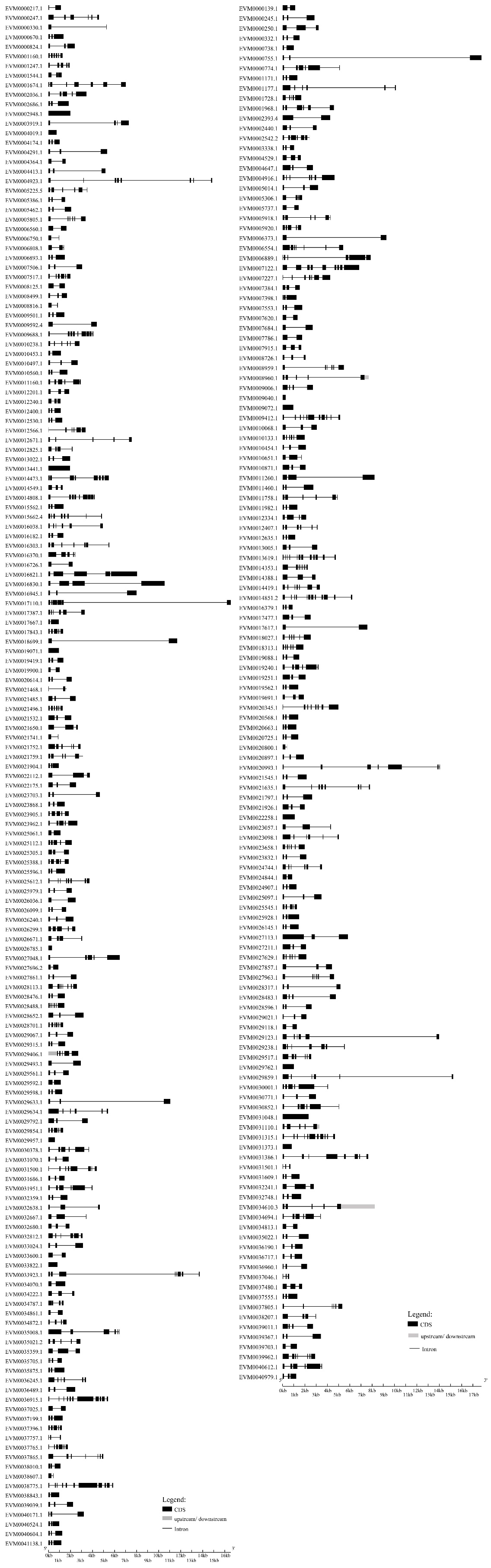


**Figure 1.** Intron/exon structure of MaMYBs.


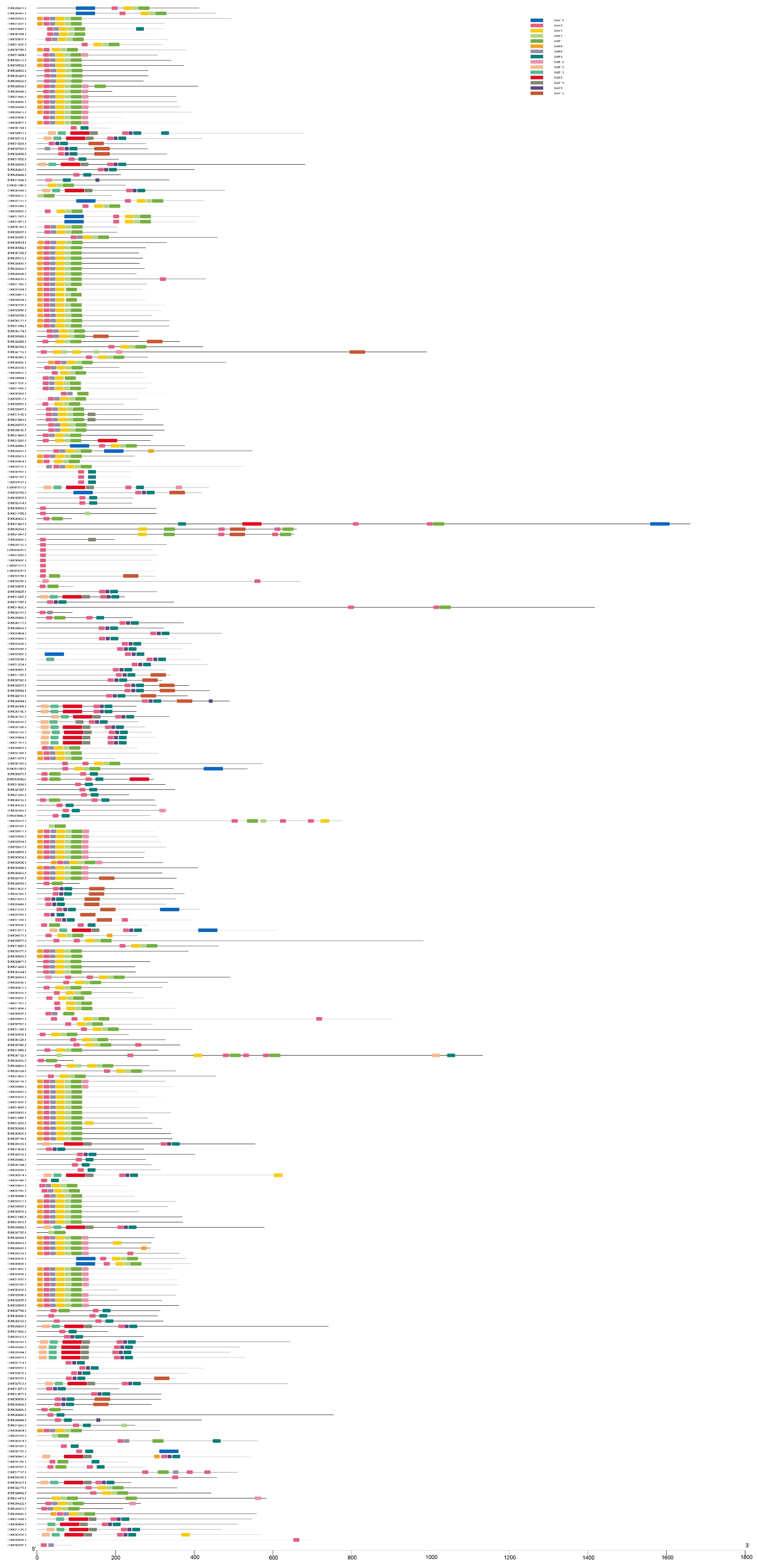


**Figure 2.** Motif structure of MaMYBs.


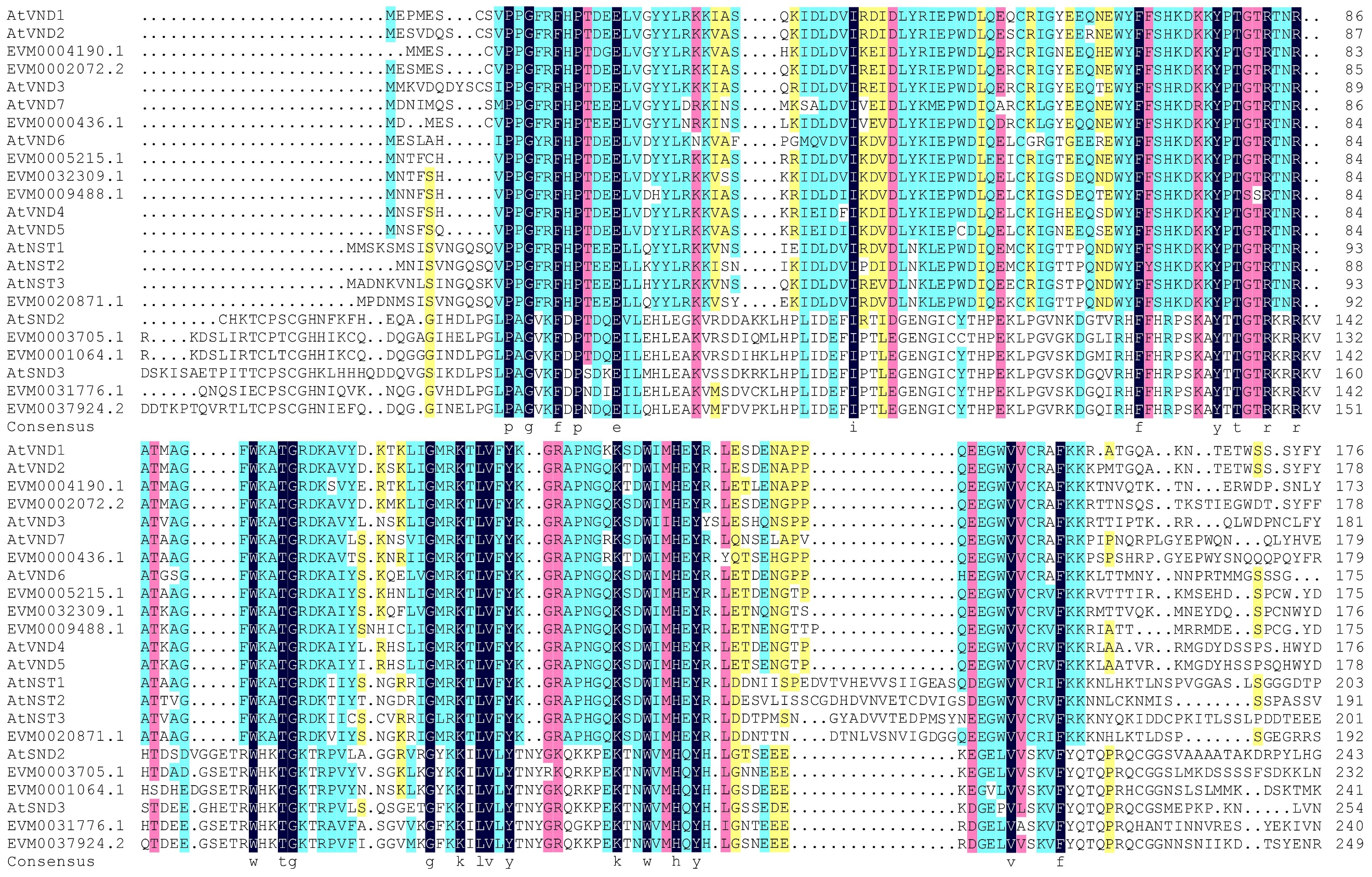


**Figure 3.** Alignment of lignin biosynthesis-related MaNACs amin acids using DNAMAN software.


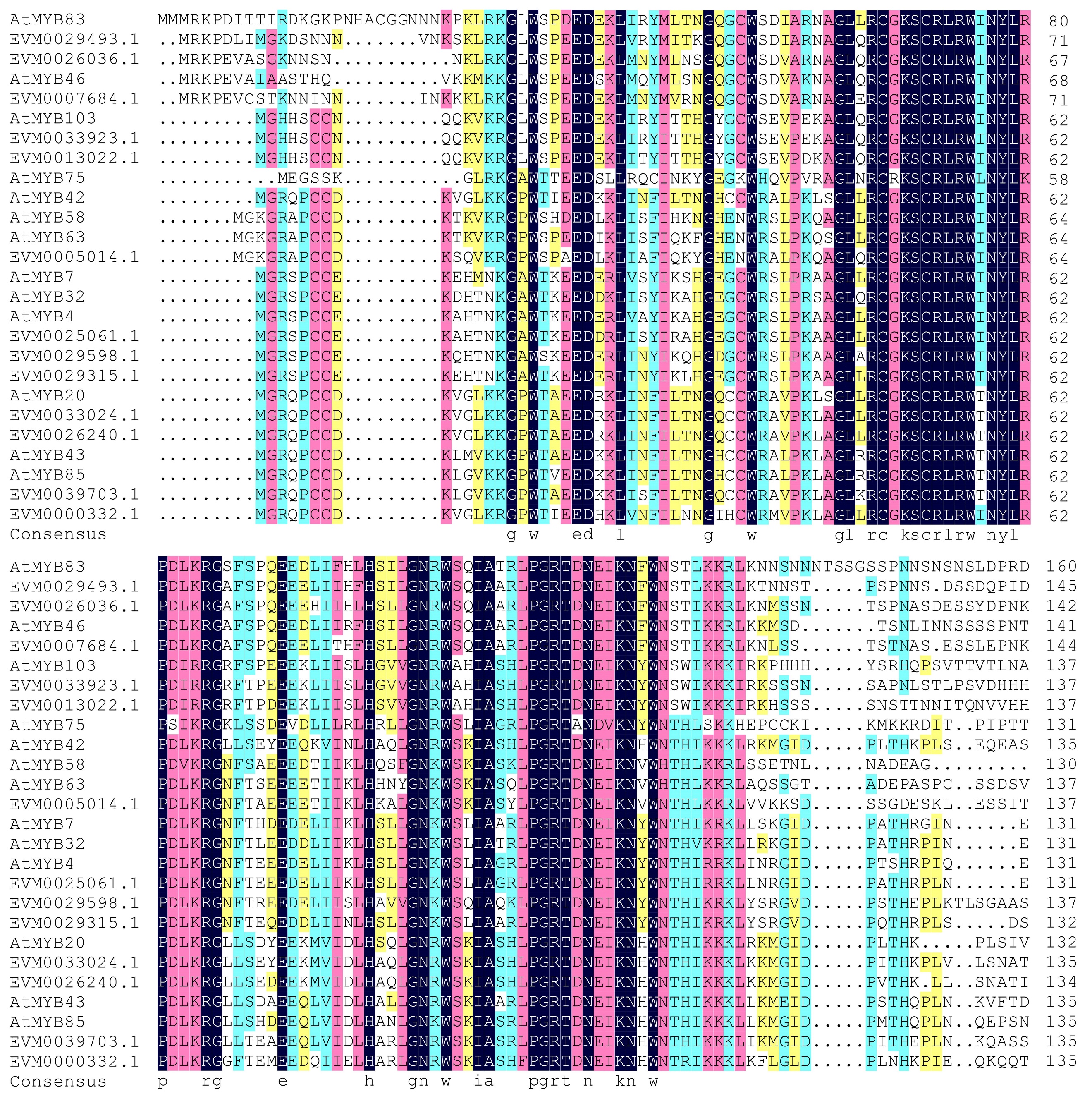


**Figure 4.** Alignment of lignin biosynthesis-related MaMYBs amin acids using DNAMAN software.


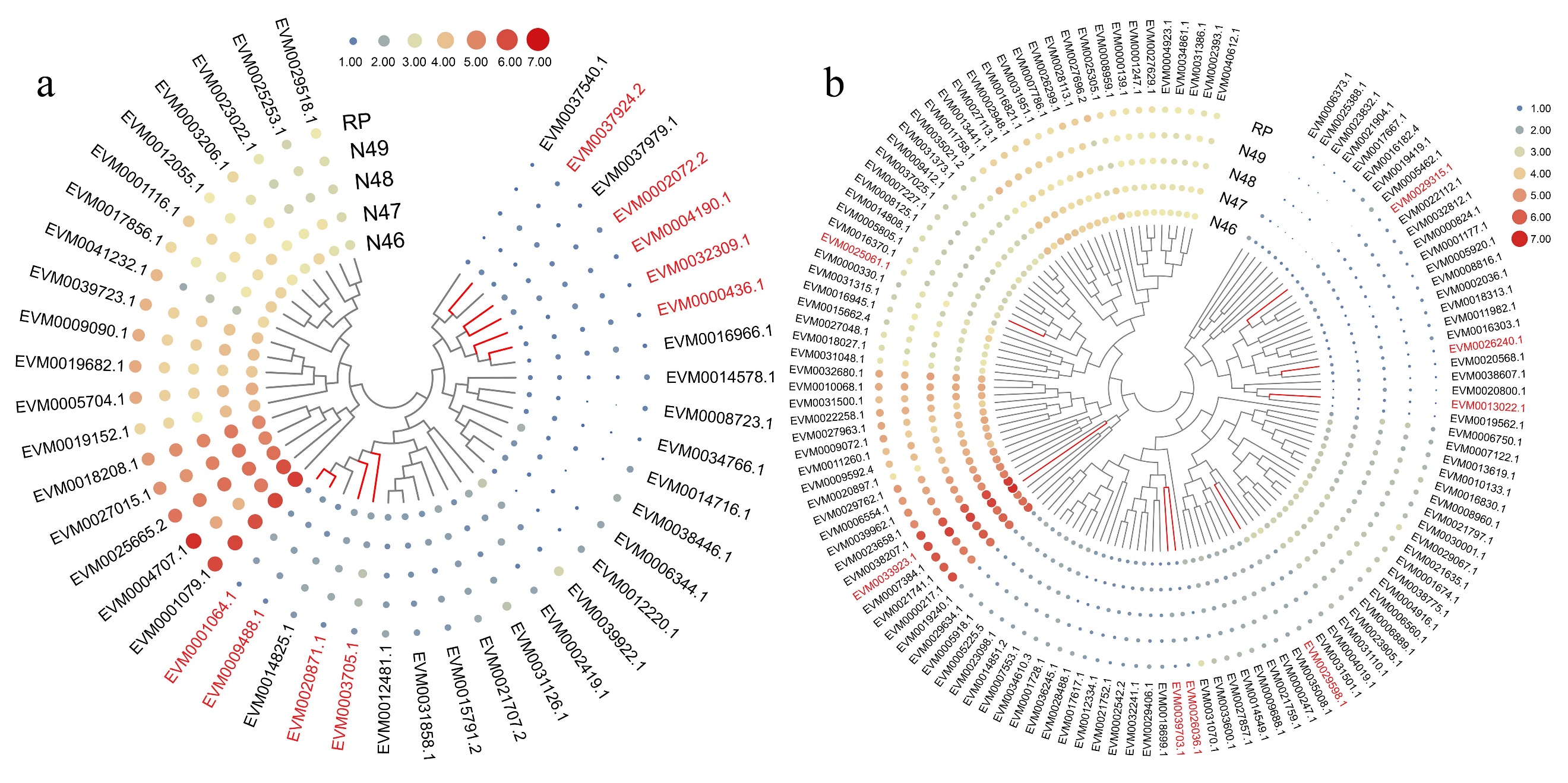


**Figure 5.** Expression profiles of *MaNAC*s and *MaMYB*s in the roots, stems, flowers, and seeds. a. Expression profiles of *MaNAC*s in the roots, stems, flowers, and seeds. b. Expression profiles of *MaMYB*s in the roots, stems, flowers, and seeds.
